# Structure and Composition of Natural Gmelin Larch (*Larix gmelinii* var. *gmelinii*) Forests in Response to Spatial Climatic Changes

**DOI:** 10.1371/journal.pone.0066668

**Published:** 2013-06-18

**Authors:** Jingli Zhang, Yong Zhou, Guangsheng Zhou, Chunwang Xiao

**Affiliations:** 1 State Key Laboratory of Vegetation and Environmental Change, Institute of Botany, the Chinese Academy of Sciences, Beijing, China; 2 University of Chinese Academy of Sciences, Beijing, China; 3 Chinese Academy of Meteorological Sciences, Beijing, China; The Ohio State University, United States of America

## Abstract

**Background:**

Many theoretical researches predicted that the larch species would decrease drastically in China under future climatic changes. However, responses of the structural and compositional changes of Gmelin larch (*Larix gmelinii* var. *gmelinii*) forests to climatic changes have rarely been reported.

**Methodology/Principal Findings:**

Field survey was conducted to examine the structures and compositions of natural Gmelin larch forests along a climatic gradient. Stepwise linear regression analyses incorporating linear and quadratic components of climatic and non-climatic factors were performed on the structural and compositional attributes of those natural Gmelin larch forests. Isothermality, Max Temperature of Warmest Month (TempWarmestMonth), Precipitation of Wettest Month (PrecipWettestMonth), Precipitation Seasonality (PrecipSeasonality) and Precipitation of Driest Quarter (PrecipDriestQuarter) were observed to be effective climatic factors in controlling structure and composition of Gmelin larch forests. Isothermality significantly affected total basal area of larch, while TempWarmestMonth, PrecipWettestMonth and PrecipSeasonality significantly affected total basal area of Mongolian pine, and PrecipDriestQuarter significantly affected mean DBH of larch, stand density of larch and total basal area of spruce and fir.

**Conclusions/Significance:**

The summer and winter temperatures and precipitations are all predicted to increase in future in Northeast China. Our results showed the increase of total basal area of spruce and fir, the suppression of regeneration and the decrease of stand density of larch under increased winter precipitation, and the decrease of total basal area of larch under increased summer temperature in the region of current Gmelin larch forest. Therefore, we suggest that larch would decrease and spruce and fir would increase in the region of future Gmelin larch forest.

## Introduction

Gmelin larch (*Larix gmelinii* var. *gmelinii*, synonym: *Larix dahurica*) is a tree species native of Russia, Mongolia and part of Northeast China [1]. In the last decade, many theoretical researches predicted that this larch species would decrease drastically in China under future climatic changes, such as the rise in mean annual temperature [2], rises in monthly temperatures [3], [4], [5] and increases in annual precipitations [2], [3]. However, responses of the structural and compositional changes of Gmelin larch forest to climatic change have rarely been reported [6], [7], [8], [9].

In order to detect and depict plant responses to large-scale climatic change, experimental studies can use spatial climatic variations instead of temporal trends, and then build linear or quadratic response functions to quantify the plants responses to climatic change. Such studies have been conducted on Lodgepole pine, grasses, tropical trees and bryophytes [10], [11], [12], [13], [14], [15], [16]. Additionally, it also is necessary to recognize the effects of some non-climatic factors, such as soil nitrogen nutrition and natural succession. Different soil nitrogen levels [17], [18] and the natural succession of community with age [19], [20], [21] can cause structural and compositional changes of communities. Usually, the stand age of natural larch forest can be represented by the maximum diameter of larch (Max.DBH_Larch_) in the stand. First, stand age is the age of the oldest tree in the stand [22], [23]. Second, the positive relationships between tree age and tree diameter are confirmed in larches [24], [25]. Although the rapidly growing tree and slowly growing tree differ in age-DBH regression slopes within the same stand, that do not make much inter-stand difference [25].

In this paper, we surveyed the structure and composition of natural Gmelin larch forests along a climatic gradient in Heilongjiang Province, Northeast China. We used multiple regression analyses to detect the effects of climatic and non-climatic factors on structure and composition of natural Gmelin larch forests. Our primary objective is to discover the climatic and non-climatic factors which control the structure and composition of Gmelin larch forests, and the second objective is to depict the responses of Gmelin larch forests to changes of those climatic and non-climatic factors.

## Materials and Methods

### Ethics Statement

All necessary permits were obtained for the described field studies. This study was approved by State Key Laboratory of Vegetation and Environmental Change, Institute of Botany, the Chinese Academy of Sciences; Forestry Administration of Mohe County (Mohe County Forestry Bureau); Huzhong National Nature Reserve; Shengshan National Nature Reserve and Liangshui National Nature Reserve.

### Study area

**Figure 1:**
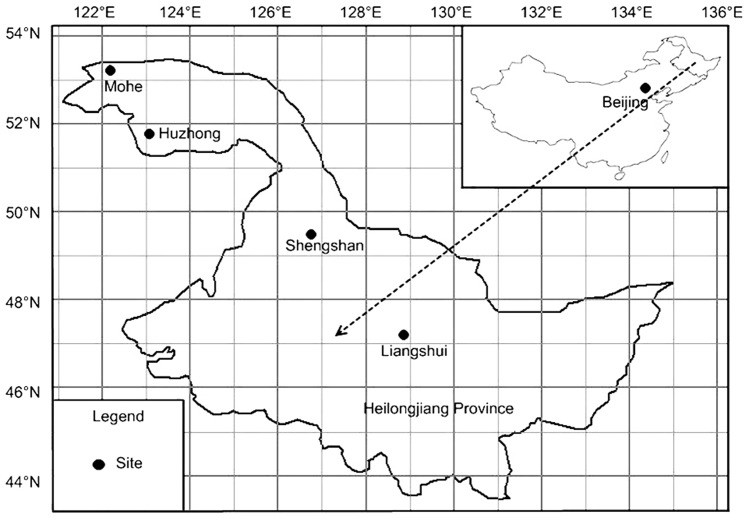
Location of study sites in Heilongjiang Province of Northeast China.

This study was conducted in July and August of 2011 along a climatic gradient in Heilongjiang Province of Northeast China, including four sites: Mohe, Huzhong, Shengshan and Liangshui (Fig. 1). Each site consists of three plots (Table 1). The forests in the plots are natural Gmelin larch forests without any evidence of recent disturbance. The region has monsoon climate: July is the warmest month and January is the coldest. Summer is the wettest quarter and winter is the driest [26], [27]. The bioclimate data of each plot downloaded from WorldClim [27] are at 1-km resolution (Table 2). Isothermality is derived as MeanDiurnalRange/TempAnnualRange, and TempAnnualRange is derived as TempWarmestMonth - TempColdestMonth. The TempWarmestQuarter, TempColdestQuarter, PrecipWarmestQuarter and PrecipColdestQuarter are exactly the same as TempWettestQuarter, TempDriestQuarter, PrecipWettestQuarter and PrecipDriestQuarter in the sites (Table 2, Table 3).

**Table 1:** Location and species of Gmelin larch forests in different sites and plots.

Site	Tree Species	Plots	Plot Latitude (°N)	Plot Longitude (°E)
Mohe	Larch (*Larix gmelinii* var. *gmelinii*), birch (*Betula platyphylla*), Mongolian pine (*Pinus sylvestris* var. *mongolica*), very few poplar (*Populus davidiana)*	Three 20 m * 20 m plots	53.2890	122.1757
			53.2889	122.1757
			53.2950	122.1978
Huzhong	Larch (*Larix gmelinii* var. *gmelinii*), birch (*Betula platyphylla*)	Three 20 m * 20 m plots	51.7808	123.0156
			51.7849	123.0164
			51.7812	123.0141
Shengshan	Larch (*Larix gmelinii* var. *gmelinii*), birch (*Betula platyphylla*)	Three 20 m * 20 m plots	49.4739	126.7462
			49.4733	126.7458
			49.4730	126.7469
Liangshui	Larch (*Larix gmelinii* var. *gmelinii*), birch (*Betula platyphylla*), spruce (*Picea koraiensis*), fir (*Abies nephrolepis*), very few Korean pine (*Pinus koraiensis*) and alder (*Alnus sibirica*)	One 40 m * 20 m plot and two 30 m * 20 m plots	47.1894	128.8815
			47.1936	128.8814
			47.1891	128.8817

**Table 2:** Variables and their abbreviations used in the study.

Variable	Abbreviation
Annual Mean Temperature (°C)	AnnualMeanTemp
Mean Diurnal Range (°C)	MeanDiurnalRange
Isothermality	Isothermality
Temperature Seasonality (standard deviation)	TempSeasonality
Max Temperature of Warmest Month (°C)	TempWarmestMonth
Min Temperature of Coldest Month (°C)	TempColdestMonth
Temperature Annual Range (°C)	TempAnnualRange
Mean Temperature of Wettest Quarter (°C)	TempWettestQuarter
Mean Temperature of Driest Quarter (°C)	TempDriestQuarter
Mean Temperature of Warmest Quarter (°C)	TempWarmestQuarter
Mean Temperature of Coldest Quarter (°C)	TempColdestQuarter
Annual Precipitation (mm)	AnnualPrecip
Precipitation of Wettest Month (mm)	PrecipWettestMonth
Precipitation of Driest Month (mm)	PrecipDriestMonth
Precipitation Seasonality (coefficient of variation)	PrecipSeasonality
Precipitation of Wettest Quarter (mm)	PrecipWettestQuarter
Precipitation of Driest Quarter (mm)	PrecipDriestQuarter
Precipitation of Warmest Quarter (mm)	PrecipWarmestQuarter
Precipitation of Coldest Quarter (mm)	PrecipColdestQuarter
Maximum DBH of Larch (cm)	Max.DBH_Larch_
0–20 cm soil nitrogen content (g/kg)	N^Soil^_0–20_
Mean DBH of Larch	MeanDBH_Larch_
Stand Density of Larch	StandDensity_Larch_
Total Basal Area of Larch	BasalArea_Larch_
Total Basal Area of Spruce and Fir	BasalArea_Spruce-Fir_
Total Basal Area of Mongolian Pine	BasalArea_MPine_
Total Basal Area of Birch	BasalArea_Birch_

**Table 3:** Climatic variables of Gmelin larch forests in different sites.

	Mohe	Huzhong	Shengshan	Liangshui
AnnualMeanTemp	–5.1±0.2	–5.3±0.0	–1.8±0.0	0.9±0.0
MeanDiurnalRange	15.2±0.0	15.7±0.0	13.5±0.0	13.1±0.0
Isothermality	0.24±0.0	0.26±0.0	0.24±0.0	0.23±0.0
TempSeasonality	168.5±0.2	162.7± 0.2	154.4±0.0	150.2±0.0
TempWarmestMonth	24.0±0.2	22.9±0.0	24.4±0.0	26.1±0.0
TempColdestMonth	–36.9±0.1	–37.2±0.0	–31.7±0.0	–29.5±0.0
TempAnnualRange	61.0±0.0	60.1±0.0	56.1±0.0	55.6±0.0
TempWettestQuarterTempWarmestQuarter	15.3±0.2	14.2±0.1	16.8±0.0	18.9±0.0
TempDriestQuarterTempColdestQuarter	–27.7±0.1	–27.4±0.0	–22.9±0.0	–19.9±0.0
AnnualPrecip	454±4	505±0	565±0	646±0
PrecipWettestMonth	112±1	135±0	148±0	164±0
PrecipDriestMonth	3±0	4±0	4±0	5±0
PrecipSeasonality	99±0	102±0	101±0	101±0
PrecipWettestQuarterPrecipWarmestQuarter	290±2	330±0	364±0	412±0
PrecipDriestQuarterPrecipColdestQuarter	15±0	15±0	14±0	19±0

Values represent mean ± standard error (n = 3). TempWarmestQuarter, TempColdestQuarter, PrecipWarmestQuarter and PrecipColdestQuarter are exactly the same as TempWettestQuarter, TempDriestQuarter, PrecipWettestQuarter and PrecipDriestQuarter in the sites.

### Field survey, soil sample collection and nitrogen measurement

In each plot, every living tree higher than 1.4 m was identified to species and measured for circumference at breast height (1.3 m).

Soil samples were collected to a depth of 20 cm using a metal cylinder (8 cm diameter and 10 cm length). In each plot, soil cores were excavated from five random points and then mixed into one sample. Fresh soil samples were passed through a 2-mm sieve to be removed of visible plant tissues, then air-dried and kept in ziplock bags.

The air-dried soil samples were taken to the State Key Laboratory of Vegetation and Environmental Change for nitrogen analyses. The 0 - 20 cm soil nitrogen contents were measured by the Semimicro-Kjeldahl method [28].

### Data analyses

The circumference of each tree was converted into tree diameter at breast height (DBH) and into tree basal area using the circle formulas. Seven attributes of Gmelin larch forest (Max.DBH_Larch_, mean DBH of larch, stand density of larch, total basal area of larch, total basal area of Mongolian pine, total basal area of spruce and fir and total basal area of birch) were extracted from each plot.

One-way ANOVA and regression and correlation analyses were performed using SPSS 13.0. Significant differences (p<0.05) between sites were detected using One-way ANOVA with post-Duncan's test. Standard errors within sites were detected by one-way ANOVA with descriptive options.

Stepwise linear regression analyses incorporating linear and quadratic components of seventeen independent factors (fifteen climatic and two non-climatic) and stepwise linear regression analyses incorporating only linear components of them were performed on six dependent variables. The fifteen climatic factors were Annual Mean Temperature (AnnualMeanTemp), Mean Diurnal Range (MeanDiurnalRange), Isothermality, Temperature Seasonality (TempSeasonality), Max Temperature of Warmest Month (TempWarmestMonth), Min Temperature of Coldest Month (TempColdestMonth), Temperature Annual Range (TempAnnualRange), Mean Temperature of Wettest Quarter (TempWettestQuarter), Mean Temperature of Driest Quarter (TempDriestQuarter), Annual Precipitation (AnnualPrecip), Precipitation of Wettest Month (PrecipWettestMonth), Precipitation of Driest Month (PrecipDriestMonth), Precipitation Seasonality (PrecipSeasonality), Precipitation of Wettest Quarter (PrecipWettestQuarter) and Precipitation of Driest Quarter (PrecipDriestQuarter), and the two non-climatic factors were Max.DBH_Larch_ and 0–20 cm soil nitrogen content (N^soil^_0–20_) (Table 2). The six dependent variables were mean DBH of larch (MeanDBH_Larch_), stand density of larch (StandDensity_Larch_), total basal area of larch (BasalArea_Larch_), total basal area of spruce and fir (BasalArea_Spruce-Fir_), total basal area of Mongolian pine (BasalArea_MPine_) and total basal area of birch (BasalArea_Birch_) (Table 2). Regression models with non-significant (p>0.05) term(s) would be rejected. Among candidate models with all terms significant (p<0.05), the model with the lowest Small Sample Unbiased Akaike Information Criterion (AICc) would be selected [29].

Two-tailed partial correlation coefficients between every included dependent variable and the independent variables excluded from its regression model were tested, controlling for the independent variable(s) included in its regression model. Pearson's two-tailed correlation coefficients among all variables were also tested.

## Results

### Gmelin larch forest structure and composition

There were significant differences in Max.DBH_Larch_, MeanDBH_Larch_, StandDensity_Larch_, BasalArea_Larch_, BasalArea_Spruce-Fir_ and BasalArea_MPine_ among the four sites (P<0.05). The MeanDBH_Larch_ roughly increased while the StandDensity_Larch_ roughly decreased from the northernmost site Mohe to the southernmost site Liangshui. BasalArea_Larch_ decreased from the second northernmost site Huzhong to the northernmost Mohe and the southernmost Liangshui. Max.DBH_Larch_ was higher in the second northernmost Huzhong and the southernmost Liangshui than that in the northernmost Mohe and the second southernmost Shengshan. Mongolian pine was observed only in the northernmost Mohe while spruce and fir were observed only in the southernmost Liangshui (Table 4).

**Table 4:** Structure and composition of Gmelin larch forests in different sites.

Site	Max.DBH_Larch_ (cm)	MeanDBH_Larch_ (cm)	StandDensity_Larch_ (No.ha^−1^)	BasalArea_Larch_ (m^2^ha^−1^)	BasalArea_Spruce-Fir_ (m^2^ha^−1^)	BasalArea_MPine_ (m^2^ha^−1^)
Mohe	28.7±3.6b	17.2±0.7b	1058±131a	27.51±4.39b	0.00±0.00b	4.36±2.24a
Huzhong	58.2±2.5a	22.0±1.8b	850±128a	44.01±2.39a	0.00±0.00b	0.00±0.00b
Shengshan	33.5±1.1b	15.7±0.3b	1142±51a	25.71±1.14bc	0.00±0.00b	0.00±0.00b
Liangshui	62.0±2.9a	42.0±9.6a	140±57b	16.38±2.69c	11.16±2.87a	0.00±0.00b

Values represent mean ± standard error (n = 3). Different letters in each column indicate significant differences among sites (post-Duncan test, P<0.05).

### Responses of structure and composition in Gmelin larch forests to climatic and non-climatic factors

Although the excluded independent variables showed no significant partial correlation with the dependent variable when controlling for the included independent variable(s) (Table 5), many of them showed significant Pearson's two-tailed correlations with not only the included independent variable(s) but also the dependent variable. AnnualMeanTemp, MeanDiurnalRange, TempWarmestMonth, TempColdestMonth, TempAnnualRange, TempWettestQuarter and TempDriestQuarter all showed significant Pearson's two-tailed correlations with Isothermality and BasalArea_Larch_. AnnualMeanTemp, TempWarmestMonth, TempWettestQuarter, TempDriestQuarter, AnnualPrecip, PrecipWettestMonth, PrecipDriestMonth, PrecipWettestQuarter and Max.DBH_Larch_ all showed significant Pearson's two-tailed correlations with PrecipDriestQuarter, MeanDBH_Larch_, StandDensity_Larch_ and BasalArea_Spruce-Fir_. TempSeasonality, PrecipDriestMonth and PrecipWettestQuarter all showed significant Pearson's two-tailed correlations with TempWarmestMonth, PrecipWettestMonth and BasalArea_MPine_ (Table 6).

**Table 5:** Matrix of two-tailed partial correlation coefficients between every included dependent variable and the independent variables excluded from its model, controlling for the independent variable(s) included in its model.

	AnnualMeanTemp	MeanDiurnalRange	Isothermality	TempSeasonality	TempWarmestMonth	TempColdestMonth	TempAnnualRange	TempWettestQuarter	TempDriestQuarter	AnnualPrecip	PrecipWettestMonth	PrecipDriestMonth	PrecipSeasonality	PrecipWettestQuarter	PrecipDriestQuarter	Max.DBH_Larch_	N^Soil^_0-20_
MeanDBH_Larch_	0.08^NS^	−0.04^NS^	0.13^NS^	−0.16^NS^	−0.06^NS^	0.09^NS^	−0.13^NS^	0.01^NS^	0.10^NS^	0.17^NS^	0.20^NS^	0.23^NS^	0.24^NS^	0.18^NS^	−	0.38^NS^	0.00^NS^
StandDensity_Larch_	−0.04^NS^	−0.05^NS^	−0.34^NS^	0.18^NS^	0.15^NS^	−0.04^NS^	0.10^NS^	0.06^NS^	−0.07^NS^	−0.18^NS^	−0.27^NS^	−0.37^NS^	−0.52^NS^	−0.22^NS^	−	−0.40^NS^	−0.07^NS^
BasalArea_Larch_	−0.24^NS^	0.18^NS^	−	0.18^NS^	−0.34^NS^	−0.23^NS^	0.18^NS^	−0.29^NS^	−0.22^NS^	−0.16^NS^	−0.17^NS^	−0.18^NS^	−0.33^NS^	−0.16^NS^	−0.08^NS^	−0.11^NS^	0.06^NS^
BasalArea_Spruce__-__Fir_	0.45^NS^	−0.44^NS^	−0.28^NS^	−0.42^NS^	0.37^NS^	0.45^NS^	−0.44^NS^	0.42^NS^	0.45^NS^	0.42^NS^	0.38^NS^	0.30^NS^	0.11^NS^	0.41^NS^	−	−0.03^NS^	0.33^NS^
BasalArea_MPine_	−0.21^NS^	0.21^NS^	0.20^NS^	0.20^NS^	−	−0.18^NS^	0.18^NS^	−0.20^NS^	−0.21^NS^	0.15^NS^	−	0.21^NS^	−	0.21^NS^	0.21^NS^	−	−0.56^NS^

“–” indicates controlling for that variable. Significance level:^ NS^ Non-significant (P>0.05), *P<0.05, **P<0.01 (n = 12).

**Table 6:** Matrix of Pearson's two-tailed correlation coefficients between variables.

	AnnualMeanTemp	MeanDiurnalRange	Isothermality	TempSeasonality	TempWarmestMonth	TempColdestMonth	TempAnnualRange	TempWettestQuarter	TempDriestQuarter	AnnualPrecip	PrecipWettestMonth	PrecipDriestMonth	PrecipSeasonality	PrecipWettestQuarter	PrecipDriestQuarter	Max.DBH_Larch_	N^Soil^_0–20_	MeanDBH_Larch_	StandDensity_Larch_	BasalArea_Larch_	BasalArea_Spruce-Fir_	BasalArea_MPine_
MeanDiurnalRange	−0.96^**^																					
Isothermality	−0.78^**^	0.82^**^																				
TempSeasonality	−0.93^**^	0.89^**^	0.52^NS^																			
TempWarmestMonth	0.93^**^	−0.88^**^	−0.93^**^	−0.73^**^																		
TempColdestMonth	0.99^**^	−0.99^**^	−0.76^**^	−0.95^**^	0.89^**^																	
TempAnnualRange	−0.94^**^	0.96^**^	0.62^*^	0.98^**^	−0.75^**^	−0.97^**^																
TempWettestQuarter	0.98^**^	−0.95^**^	−0.88^**^	−0.84^**^	0.98^**^	0.96^**^	−0.87^**^															
TempDriestQuarter	1.00^**^	−0.96^**^	−0.73^**^	−0.96^**^	0.89^**^	0.99^**^	−0.96^**^	0.96^**^														
AnnualPrecip	0.95^**^	−0.87^**^	−0.57^NS^	−0.98^**^	0.79^**^	0.94^**^	−0.93^**^	0.88^**^	0.97^**^													
PrecipWettestMonth	0.88^**^	−0.80^**^	−0.40^NS^	−0.98^**^	0.66^*^	0.88^**^	−0.92^**^	0.77^**^	0.92^**^	0.98^**^												
PrecipDriestMonth	0.82^**^	−0.68^*^	−0.32^NS^	−0.91^**^	0.63^*^	0.79^**^	−0.80^**^	0.72^**^	0.85^**^	0.95^**^	0.97^**^											
PrecipSeasonality	0.22^NS^	−0.11^NS^	0.43^NS^	−0.52^NS^	−0.11^NS^	0.24^NS^	−0.39^NS^	0.04^NS^	0.29^NS^	0.47^NS^	0.63^*^	0.66^*^										
PrecipWettestQuarter	0.93^**^	−0.85^**^	−0.52^NS^	−0.98^**^	0.75^**^	0.92^**^	−0.93^**^	0.85^**^	0.96^**^	1.00^**^	0.99^**^	0.96^**^	0.52^NS^									
PrecipDriestQuarter	0.70^*^	−0.50^NS^	−0.57^NS^	−0.56^NS^	0.78^**^	0.59^*^	−0.45^NS^	0.73^**^	0.67^*^	0.71^**^	0.61^*^	0.74^**^	0.10^NS^	0.69^*^								
Max.DBH_Larch_	0.35^NS^	−0.11^NS^	0.13^NS^	−0.47^NS^	0.20^NS^	0.27^NS^	−0.28^NS^	0.25^NS^	0.37^NS^	0.57^NS^	0.62^*^	0.78^**^	0.67^*^	0.60^*^	0.67^*^							
N^Soil^_0–20_	0.65^*^	−0.79^**^	−0.42^NS^	−0.75^**^	0.43^NS^	0.75^**^	−0.85^**^	0.57^NS^	0.69^*^	0.63^*^	0.66^*^	0.45^NS^	0.29^NS^	0.63^*^	−0.03^NS^	−0.11^NS^						
MeanDBH_Larch_	0.61^*^	−0.43^NS^	−0.41^NS^	−0.54^NS^	0.62^*^	0.53^NS^	−0.44^NS^	0.61^*^	0.59^*^	0.65^*^	0.59^*^	0.70^*^	0.22^NS^	0.64^*^	0.82^**^	0.71^**^	−0.03^NS^					
StandDensity_Larch_	−0.66^*^	0.45^NS^	0.42^NS^	0.57^NS^	−0.68^*^	−0.56^NS^	0.45^NS^	−0.66^*^	−0.64^*^	−0.71^*^	−0.64^*^	−0.78^**^	−0.29^NS^	−0.70^*^	−0.93^**^	−0.73^**^	0.01^NS^	−0.86^**^				
BasalArea_Larch_	−0.78^**^	0.80^**^	0.92^**^	0.54^NS^	−0.91^**^	−0.76^**^	0.63^*^	−0.87^**^	−0.73^**^	−0.58^*^	−0.43^NS^	−0.37^NS^	0.28^NS^	−0.53^NS^	−0.55^NS^	0.08^NS^	−0.37^NS^	−0.49^NS^	0.50^NS^			
BasalArea_Spruce-Fir_	0.77^**^	−0.62^*^	−0.61^*^	−0.66^*^	0.80^**^	0.69^*^	−0.58^*^	0.79^**^	0.75^**^	0.77^**^	0.68^*^	0.75^**^	0.14^NS^	0.75^**^	0.90^**^	0.59^*^	0.11^NS^	0.80^**^	−0.84^**^	−0.61^*^		
BasalArea_MPine_	−0.36^NS^	0.33^NS^	−0.10^NS^	0.61^*^	−0.06^NS^	−0.39^NS^	0.53^NS^	−0.21^NS^	−0.42^NS^	−0.57^NS^	−0.66^*^	−0.63^*^	−0.59^*^	−0.60^*^	−0.17^NS^	−0.58^*^	−0.50^NS^	−0.26^NS^	0.16^NS^	−0.22^NS^	−0.24^NS^	
BasalArea_Birch_	0.33^NS^	−0.25^NS^	−0.26^NS^	−0.27^NS^	0.36^NS^	0.29^NS^	−0.23^NS^	0.34^NS^	0.32^NS^	0.32^NS^	0.28^NS^	0.32^NS^	0.07^NS^	0.31^NS^	0.41^NS^	0.36^NS^	−0.05^NS^	0.66^*^	−0.39^NS^	−0.40^NS^	0.38^NS^	−0.06^NS^

Significance level:^ NS^ Non-significant (P>0.05), *P<0.05, **P<0.01 (n = 12).

Significant regression models were observed for the dependent variables except for BasalArea_Birch_. For total basal area of Mongolian Pine, the model with 5 variables was selected because it showed the lowest AICc value (–17.25) among all candidate models. Isothermality had positive effect on BasalArea_Larch_. PrecipDriestQuarter had positive effect on MeanDBH_Larch_ and BasalArea_Spruce-Fir_, and negative effect on StandDensity_Larch_. TempWarmestMonth and PrecipSeasonality had positive effects while PrecipWettestMonth had negative effect on BasalArea_MPine_. Max.DBH_Larch_ was observed to have negative relationship with BasalArea_MPine_ when it was below 63.25 cm (Table 7).

**Table 7:** Significant mathematical equations and regression coefficients (a, b, c, d, e, f) used to predict structure and composition of Gmelin larch forests from linear and quadratic components of Isothermality, TempWarmestMonth, PrecipWettestMonth, PrecipSeasonality, PrecipDriestQuarter and Max.DBH_Larch_.

Dependent Variable, Y	Model Form	Coefficients						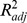
		a (S.E.)	b (S.E.)	c (S.E.)	d (S.E.)	e (S.E.)	f (S.E.)	
MeanDBH_Larch_	Y = a+b(PrecipDriestQuarter)	−60.8(18.8)^**^	5.4 (1.2)^**^	−	−	–		0.64^**^
StandDensity_Larch_	Y = a+b(PrecipDriestQuarter)^2^	2316.1 (203.0)^***^	-6.0 (0.8)^***^	–	–	–		0.84^***^
BasalArea_Larch_	Y = a+b(Isothermality)	−192.3(29.4)^***^	910.2(121.1)^***^	–	–	–		0.84^***^
BasalArea_Spruce-Fir_	Y = a+b(PrecipDriestQuarter)^2^	−15.8 (2.8)^***^	0.08 (0.01)^***^	–	–	–		0.80^***^
BasalArea_MPine_	Y = a+b(TempWarmestMonth)+c(PrecipWettestMonth)+d(PrecipSeasonality)+e(Max.DBH_Larch_)+f(Max.DBH_Larch_)^2^	−347.8 (18.6)^***^	4.0 (0.2)^***^	−0.34 (0.01)^***^	3.04 (0.16)^***^	−0.25(0.05)^**^	0.002 (0.001)^**^	0.99^***^

Equations from stepwise regression analyses (n = 12). S.E., standard error; 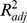, adjusted multiple coefficient of determination. Significance level:^ NS^ Non-significant (P>0.05), *P<0.05, **P<0.01, ***P<0.001.

## Discussion

In Daxing'anling Mountains of China, the importance value of Mongolia pine decreases while the importance value of larch increases from the 19th year to the 100th year after fire disturbance in the succession layer of Gmelin larch forest [30]. In our result, the Max.DBH_Larch_ was observed to have significant negative relationship with total basal area of Mongolian pine (given that the Max.DBH_Larch_ in Mohe didn't exceed 63.25 cm), but no significant relationship with total basal area of larch (Table 6, Table 7). As the Max.DBH_Larch_ represents the stand age, we suggest the natural decline of Mongolian pine in Gmelin larch forest with increasing stand age.

Spruce (*Picea spp.*) and fir (*Abies spp.*) are more shade-tolerant than larch [31], [32]. In the absence of spruce and fir in northeastern China, larch can maintain its canopy dominance via gap regeneration [33]. But when larch coexists with those more shade-tolerant spruce and fir, larch will not maintain itself under the canopy, and will be ultimately replaced by those more tolerant and self-maintaining evergreens (spruce and fir) [34], [35]. Exposure to direct sunlight can cause winter injuries to conifers such as spruce [36]. The exposed conifer seedlings show more serious winter injuries and increase mortality compared with snow-covered seedlings [37], [38]. However, the beneficial effects of snow cover (for example on dry matter productions) are more pronounced on spruce than on larch [39], while the larch has a higher resistance to winter injuries than fir has [40]. In our results, PrecipDriestQuarter was observed to have positive effect on total basal area of spruce and fir, negative effect on stand density of larch and positive effect on mean DBH of larch. As PrecipDriestQuarter is winter precipitation [26] (Table 3), a higher PrecipDriestQuarter can form a thicker snow-cover, protecting the evergreen conifer seedlings (spruce and fir) from winter injury and mortality, resulting in more evergreen conifers (spruce and fir) in Gmelin larch forest. The negative relationship between PrecipDriestQuarter and stand density of larch and the positive relationship between PrecipDriestQuarter and mean DBH of larch indicated that larch regeneration is suppressed in presence of those evergreen conifers (spruce and fir), resulting in a lower density of larch and a lack of younger (smaller) larch stems.

The positive effects of summer temperatures on radial growths of larch were observed in polar and alpine treelines (ecotones between forest and tundra) [6], [7], but the negative effects of July temperatures on radial growths of larch were observed in China [41], [42] and Central Yakutia [9]. In our results, the positive effect of Isothermality on total basal area of larch was observed. Thus, a higher TempWarmestMonth (hotter July) can cause a lower Isothermality, and result in a decrease in total basal area of larch. Although a higher TempColdestMonth can cause a higher Isothermality, the correlation analyses suggested the negative effect of TempColdestMonth on total basal area of larch instead of positive (Table 6).

Some studies found the negative effects of summer temperatures and positive effects of summer precipitations on radial growths of Mongolian pine (*Pinus sylvestris* var. *mongolica*) in China [43], [44], [45]. However, a hotter and dryer summer do more harm to larch than to pine (*Pinus sylvestris*), causing a superiority of pine in competition with larch [46]. In our study, the positive effect of TempWarmestMonth and negative effect of PrecipWettestMonth on total basal area of Mongolian pine were observed, suggesting that a hot and dry summer may suppress larch and favor the regeneration of Mongolian pine in Gmelin larch forest.

Most of the excluded independent variables showed some significant correlations with the dependent variables, and every excluded independent variable showed some significant correlation(s) with the included independent variable(s) (Table 6). The significant correlations indicated the redundancy of independent variables. Further research will be needed to tell whether the excluded independent variables are also effective in controlling structure and composition of Gmelin larch forests.

The summer and winter temperatures and precipitations in Northeast China will all increase in the 21st century under the SRES A1B, A2, B1 and B2 scenarios, as predicted by the CCCMA-GCM 3.1, CNRM-CM3, MPI-CHAM 5, UKMO-HadCM 3 and PRECIS models [47], [48]. Based on the climatic predictions, our results suggested the decrease of total basal area and stand density of larch and the increase of total basal area of spruce and fir in the Gmelin larch forest in future. Many studies also predicted the decrease of Gmelin larch [2], [3] or decrease of larch [4], [5] in northeastern China under increased temperatures and precipitations. However, some studies predicted the larch would be replaced by pine and broad-leaved trees [4], [5], while our study predicted the larch would be replaced by spruce and fir. Further research will be needed to tell which tree species will replace Gmelin larch and whether the total basal area of Mongolia pine will increase or decrease in future.

## Conclusions

The summer and winter temperatures and precipitations are all predicted to increase in future in Northeast China [47], [48]. Our results showed the increase of total basal area of spruce and fir, the suppression of regeneration and the decrease of stand density of larch under increased winter precipitation, and the decrease of total basal area of larch under increased summer temperature in the region of current Gmelin larch forest. Therefore, we suggest that larch would decrease and spruce and fir would increase in the region of future Gmelin larch forest.
